# Advances in Microfluidic Systems and Numerical Modeling in Biomedical Applications: A Review

**DOI:** 10.3390/mi15070873

**Published:** 2024-06-30

**Authors:** Mariana Ferreira, Violeta Carvalho, João Ribeiro, Rui A. Lima, Senhorinha Teixeira, Diana Pinho

**Affiliations:** 1 Center for Microelectromechanical Systems (CMEMS-UMinho), University of Minho, Campus de Azurém, 4800-058 Guimaraes, Portugal; pg51212@uminho.pt (M.F.); diana.pinho@cmems.uminho.pt (D.P.); 2 LABBELS—Associate Laboratory, 4800-058 Guimaraes, Portugal; st@dps.uminho.pt; 3 MEtRICs, Mechanical Engineering Department, University of Minho, Campus de Azurém, 4800-058 Guimaraes, Portugal; rl@dem.uminho.pt; 4 ALGORITMI Center/LASI, University of Minho, Campus de Azurém, 4800-058 Guimaraes, Portugal; 5 Instituto Politécnico de Bragança, 5300-052 Bragança, Portugal; jribeiro@ipb.pt; 6 Laboratório Associado para a Sustentabilidade e Tecnologia em Regiões de Montanha (SusTEC), Campus Santa Apolónia, 5300-253 Bragança, Portugal; 7 CIMO—Mountain Research Center, Campus Santa Apolónia, 5300-253 Bragança, Portugal; 8 CEFT—Transport Phenomena Research Center, Faculty of Engineering, University of Porto, Rua Dr. Roberto Frias, 4200-465 Porto, Portugal; 9 ALiCE—Associate Laboratory in Chemical Engineering, Faculty of Engineering, University of Porto, Rua Dr. Roberto Frias, 4200-465 Porto, Portugal

**Keywords:** numerical simulation, microfluidics, organ-on-a-chip, microfluidics systems, mixing

## Abstract

The evolution in the biomedical engineering field boosts innovative technologies, with microfluidic systems standing out as transformative tools in disease diagnosis, treatment, and monitoring. Numerical simulation has emerged as a tool of increasing importance for better understanding and predicting fluid-flow behavior in microscale devices. This review explores fabrication techniques and common materials of microfluidic devices, focusing on soft lithography and additive manufacturing. Microfluidic systems applications, including nucleic acid amplification and protein synthesis, as well as point-of-care diagnostics, DNA analysis, cell cultures, and organ-on-a-chip models (e.g., lung-, brain-, liver-, and tumor-on-a-chip), are discussed. Recent studies have applied computational tools such as ANSYS Fluent 2024 software to numerically simulate the flow behavior. Outside of the study cases, this work reports fundamental aspects of microfluidic simulations, including fluid flow, mass transport, mixing, and diffusion, and highlights the emergent field of organ-on-a-chip simulations. Additionally, it takes into account the application of geometries to improve the mixing of samples, as well as surface wettability modification. In conclusion, the present review summarizes the most relevant contributions of microfluidic systems and their numerical modeling to biomedical engineering.

## 1. Introduction

The relentless pursuit of advancements in the field of biomedical engineering has led to the exploration of new technologies and approaches that could revolutionize disease diagnosis, treatment, and monitoring. In this context, microfluidic systems are emerging as a promising revolution, offering an interdisciplinary research field that combines principles of physics, engineering, and biology to create microscale devices enabling the precise manipulation of biological fluids. These systems have the potential to radically transform medicine by providing innovative solutions to complex biomedical challenges [1,2].

Microfluidic devices are becoming more and more important in several applications, from scientific to industrial areas. This exponential interest in microfluidic systems is more notable in the last 20 years, where this development has also substantial interest in biomedical engineering applications [3,4]. When talking about the biomedical engineering area, one of the most important advances is in microfluidic systems dedicated to diagnosis applications [5,6], point-of-care devices [7,8], drug delivery [9], cell culture in OoC devices [10], biosensors [11], and wearable and implantable devices [12,13]. Recent progress in small electronic devices, when used with biological sensors and microheaters, enables precise temperature and real-time monitoring, helping us to better understand how our cells work. These functionalities are essential for applications such as nucleic acid amplification and enzyme-linked immunosorbent assays (ELISA) for protein detection. Continuous health monitoring benefits from such capabilities by enabling the long-term tracking of biomarkers and physiological parameters, facilitating early disease detection, as well as personalized medicine approaches [14]. Essentially, these systems usually offer better performance in analysis, combine many functions, are easier to automate and control, use fewer substances, are safer, cost less, are more sensitive, and provide faster results compared to larger conventional systems [4]. Microfluidic devices are useful systems in terms of reactions, separations, and the detection of different compounds. It can also be helpful not only in devices with microreactors, LoC, or OoC but also in daily applications from pregnancy at-home testing to virus rapid testing and blood glucose monitoring [15].

In the last few years, numerical simulations have emerged as an indispensable tool in research and for the development of microfluidic systems [2]. The facility to model and predict the behavior of fluids at the microscale, within channels and devices of microlevel dimensions, has profound implications in various fields of science and technology. The complex and multiphase dynamics observed in microfluidic systems, coupled with the growing demand for efficiency and precision in biomedical, chemical, and industrial applications, have stimulated the rapid advancement of numerical simulation techniques in this field [16].

Even knowing that there are a substantial number of review papers in the microfluidic field, the number of review papers about approaches using numerical simulations as a tool is low [2,17,18]. Taking into account this gap, the goal of this work is to provide a comprehensive overview of the approaches, methods, and recent advances of both the numerical simulations of microfluidic systems and advancements in microfluidic systems for applications in biomedical engineering. On the one hand, the study tries to comprehend the limitations and particularities of the numerical simulations of microfluidic systems by analyzing several studies in this area. On the other hand, by combining numerical simulation with microfluidic systems, this review offers an embracing and innovative view of the field. This work aims to fill this gap by providing a comprehensive review of this subject and combining both topics.

## 2. Microfluidic Systems for Biomedical Engineering Applications

### 2.1. Theoretical Principles of Microfluidics

Microfluidic devices often operate under laminar flow conditions due to their small dimensions. In laminar flow, the fluid flows in parallel layers with minimal mixing between adjacent layers. Flow velocities are relatively low, and fluid particles move along predictable paths. Laminar flow is governed by viscous forces, and turbulence is absent or minimal. Understanding laminar flow is crucial for designing efficient microfluidic systems for various applications [19,20].

The Reynolds number (Re) is a dimensionless parameter that characterizes the flow regime. In microfluidics, the Re is typically very low due to small channel dimensions and low flow rates. Laminar flow occurs at Re values below a critical threshold, typically around 2000 [21,22]. Microfluidic channels are often characterized by low aspect ratios (width-to-height ratio), promoting laminar flow. Surface effects, such as channel wall interactions, play a significant role in determining flow behavior [23]. Laminar flow enables precise control over particle manipulation within microchannels. Techniques like inertial focusing and deterministic lateral displacement rely on laminar flow principles to separate and manipulate particles based on size or other characteristics [24].

The Navier–Stokes equations represent a mathematical formulation of Newton’s second law of motion applied to fluid flow, and they are widely used to model a variety of fluid dynamics phenomena [25]. The Navier–Stokes equations, in their full form, are represented in Equation (1) [25,26]:

(1) $$\rho \left(\frac{\partial v}{\partial t}+v\nabla v\right)=\nabla p+\mu \nabla T+f$$

where v represents the velocity vector field of the fluid, t is time, p is pressure, ρ is density, μ is the kinematic viscosity of the fluid, ∇ is the gradient operator, T is the stress and f represents the body forces per unit mass acting on the fluid.

It is proven that by using Navier–Strokes equations, analytical solutions can only be obtained for simple laminar flows. Knowing this limitation, normally computation fluid dynamics (CFD), for example, can be used to numerically solve the Navier–Strokes equation, showing to be successful in various fluid dynamic problems. After all, there are also many limitations and challenges in using CFD for complex flows [27].

The Poiseuille law describes a fundamental formulation in fluid dynamics, particularly in systems like blood vessels, given by Equation (2) [28]:

(2) $$Q=\frac{\Delta {P\pi r}^{4}}{8\eta L}$$

where Q is the volumetric flow rate of the fluid (volume per unit time), ΔP is the pressure difference between the sides of the tube, r is the internal radius of the tube, η is the dynamic viscosity of the fluid, and L is the length of the tube.

Poiseuille’s law is applicable only in laminar flow and to Newtonian fluids, which are fluids with a constant viscosity that do not change with the rate of deformation. For tubes with variable geometries, the analysis is more complex, and Poiseuille’s law may not be applicable [28]. Diffusion is the movement of molecules from a region of higher concentration to a region of lower concentration. It is a fundamental transport mechanism in microfluidic devices, in particular for mixing processes where molecular transport occurs by concentration gradients. Diffusion is described by Fick’s laws. Fick’s first law states that the diffusion flux is proportional to the concentration gradient, indicating the direction and magnitude of molecular transport [29]. The second Fick’s law describes how the concentration of molecules changes over time, considering both time and spatial variables [29].

In microfluidic devices, diffusion is particularly effective due to the small dimensions of the channels, reducing the distance over which molecules need to flow, resulting in rapid mixing and reaction kinetics [30]. Microfluidic platforms often exploit diffusion for applications like gradient generation, molecular separation, biochemical assays, cell culture, or drug testing [30].

Overall, Poiseuille’s law and diffusion are key principles that enable the precise control and manipulation of fluids and molecules in microfluidic systems, paving the way for advancements in biomedical and chemical applications.

### 2.2. Techniques to Manufacture Microfluidic Devices

Certainly, among academic researchers, polydimethylsiloxane (PDMS) stands out as the most commonly employed mineral–organic polymer for manufacturing microfluidic channels, and soft lithography is the most prevalent fabrication method. Soft lithography offers the flexibility to achieve microfluidic patterns with precision spanning from nanometers to micrometers, contingent upon the resolution of the initial master mold. Traditionally, the master mold is produced through standard photolithography procedures. In this case, a negative photoresist is meticulously applied to a silicon wafer to form microchannel projections. While photolithography consistently delivers master molds of outstanding resolution and supports the rapid development of PDMS chips, it has a notable drawback. Its dependence on expensive cleanroom facilities reduces the cost-effectiveness, especially in regions with limited resources and developing economies [31].

In response to these challenges, various endeavors have been undertaken to reduce costs and streamline the process. These efforts comprehend alternative techniques like additive manufacturing and diverse lithographic methods. Notably, additive manufacturing, popularly known as 3D printing, has emerged as a promising possibility [18]. It allows for the direct fabrication of master molds with relatively high resolution, obviating the need for intricate cleanroom setups. This approach presents a cost-effective alternative, particularly advantageous for low-cost and expeditious prototyping of microfluidic devices [32]. Among all additive manufacturing technologies, the most common techniques for producing microfluidic devices are described in the following sections, and their advantages are summarized in Table 1.

#### 2.2.1. Stereolithography (SLA)

This technology is particularly known for its ability to produce highly detailed and intricate structures with a high level of accuracy. The process involves the use of a photosensitive liquid resin that solidifies when exposed to a specific wavelength of light. In the field of microfluidics, SLA offers advantages by eliminating the need for complex steps and equipment associated with conventional technologies. Different resins, such as clear, model, tough, amber, and dental, can be used. Each resin has unique properties and behaviors, and its suitability for specific applications has been studied [33,34]. Anthony K. Au et al., 2014 [35], evaluated the feasibility of using SLA for manufacturing microfluidic devices compared to traditional PDMS molding and plastic molding techniques. They highlighted that, although PDMS molding is common due to its suitability for prototyping and physicochemical properties for biomedical and scientific applications, it is a slow process and not ideal for large-scale production. Likewise, large-scale plastic molding is expensive, making it difficult for small startups to commercialize. In contrast, SLA is an automated technique that allows the production of almost arbitrary 3D shapes in a single polymeric material with medium-scale production. Devices manufactured using SLA can be designed using computer-aided design (CAD) tools. The study evaluated the resolution, optical clarity, and commercial potential of SLA for microfluidic devices, discussing its advantages and challenges [35].

#### 2.2.2. Fused Deposition Modeling (FDM)

This is a technology used to create 3D objects layer-by-layer. In the fabrication of microfluidic devices, specifically for the development of integrated, transparent, and sealed microchannels made with thermoplastic materials, such as polylactic acid (PLA), they are heated and extruded layer-by-layer to form a three-dimensional object. One of the advantages of FDM is the ability to integrate various materials within the microchannel structure, such as paper, glass, wire, and polymers [36]. G. Gaal et al., 2017 [36], explored the use of PDMS-based microfluidic devices due to their properties of transparency, flexibility, and ability to reversibly and irreversibly adhere to different materials. However, due to the cost and implications of PDMS, an alternative was sought. Three-dimensional printing technology, specifically FDM, was employed to produce integrated, transparent, and sealed microchannels made from polylactic acid, a more affordable material. Using a homemade 3D printer, it was possible to assemble devices in a simplified way. To demonstrate the effectiveness of this method, a 3D-printed electronic tongue sensor was built, capable of distinguishing basic flavors below human limits. This approach opens up new possibilities for developing more affordable and versatile microfluidic systems [36].

#### 2.2.3. Selective Laser Melting (SLM)

SLM is an additive manufacturing technique that utilizes a laser to melt layers of metal powder, layer-by-layer, to build three-dimensional objects. Although SLM is most commonly associated with the manufacturing of metal parts, it can also be applied to the fabrication of microfluidic devices. SLM is capable of producing very small details and complex geometries with high precision, which is crucial for manufacturing microfluidic devices that often require channels, microcavities, and other intricate features. SLM can produce prototypes of microfluidic devices quickly and efficiently, allowing for iterative design cycles for product optimization [37]. N. Zhang et al., 2019 [38], developed a new method for fabricating molds for injection molding of thermoplastic microfluidic chips suitable for prototyping and early-stage scale-up. They used this additive manufacturing technique, SLM, to manufacture these molds. In the process, micrometallic patterns were printed onto a pre-finished substrate to form a microstructured mold. The researchers characterized dimensional accuracy, surface morphology, bonding strength between the printed patterns and substrate, as well as the microstructure of microfeatures. A microfluidic mold was successfully printed and used directly for injection molding of cyclic olefin copolymer microfluidic chips, which were subsequently used to monitor nitrite concentrations in environmental water. Characterization of the produced molds indicated that this new process could be used for the fast manufacturing of mold tools for injection molding/hot-embossing microfluidic devices. It is faster, more flexible, and less expensive than conventional micromachining processes, although its accuracy and finish still need improvement through process optimization and hybrid SLM and machining processes [38].

### 2.3. Materials in Microfluidic Devices

In the initial phase, glass and silicon were the most used materials in these types of studies [39,40]. While glass and silicon technologies provide excellent precision, the processes involved in making them are intricate, time-consuming, and expensive due to the need for cleanroom facilities for each device. Moreover, both glass and silicon are fragile and not suitable for disposable devices due to their high cost. It is worth mentioning that silicon is also not suitable for certain separation and detection methods because it is optically opaque and behaves like a semiconductor, which can pose risks of sample carryover and cross-contamination [41,42]. At that point, a big necessity to find new materials that supply all necessities started, which led to the use of alternative materials such as polymers. Polymers are materials with numerous benefits, including affordability, strong mechanical and chemical properties, flexibility, and straightforward processing [43]. In microfluidic applications, some commonly used polymers include PDMS, poly (methyl methacrylate) (PMMA), high-density polyethylene (HDPE), low-density polyethylene (LDPE), polyamide 6, and SU-8 (see Table 2) [44].

PDMS is one of the most popular materials to fabricate microfluidic channels. It is a type of elastomer with advantageous properties for creating microstructures in various applications, primarily due to its cost-effectiveness. Additionally, this polymer exhibits adequate thermal stability, low surface tension, and transparency for wavelengths in the range of 290 to 1100 nm. It is non-flammable and non-toxic, serving as an excellent electrical and thermal insulator, and is inert to many chemicals. Despite its ability to resist high temperatures, exposure to ultraviolet light, and ozone ($O_{3}$), PDMS degrades completely and rapidly in natural environments, posing no environmental concerns [52].

However, PDMS’s natural surface is hydrophobic due to the presence of methyl groups (${\mathrm{CH}}_{3}$), which can be a drawback in microfluidic applications, hindering its interaction with aqueous solvents and promoting water bubble retention. In biological contexts, this hydrophobicity can make surfaces susceptible to non-specific protein binding, potentially causing issues. Nevertheless, this hydrophobicity can be controlled [53]. Other relevant characteristics of PDMS include its permeability, which is determined by the solubility of gases in the polymer and the rate of gas diffusion. PDMS’s remarkable elasticity is a result of its highly coiled conformation, directly influenced by the degree of cross-linking; the higher the cross-linking, the lower the elasticity [54].

### 2.4. Microfluidic Systems Applications

#### 2.4.1. Diagnostic Applications

**Figure 1:**
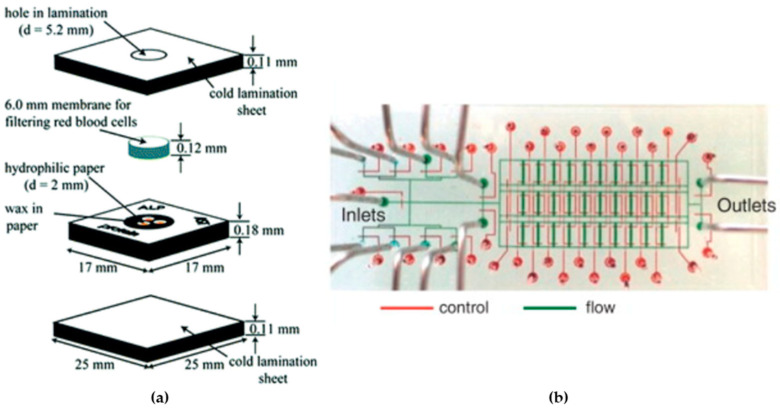
Diagnostic applications: (**a**) design of a flow-through device (adapted from [55]), and (**b**) integrated microfluidic device fabricated by soft lithography (obtained from [62]).

Microfluidic devices emerge as a promising technology for point-of-care diagnostics, providing to the growing demand for economically viable diagnostic solutions (see Figure 1a). However, the translation of these diagnostics to developing regions encounters obstacles related to design intricacies, cost-effectiveness, and manufacturing complexities. These devices could prove beneficial in specific point-of-care scenarios with more readily available resources compared to resource-scarce settings, where the affordability of the diagnostic tool is a lesser constraint. Such microfluidic devices share similarities with existing lateral-flow assays, yet their microfluidic elements introduce the prospect of enhanced functionalities beyond what has been conventionally accessible [55].

Point-of-care testing (POCT) devices have become more advanced, using microheaters and microsensors to improve the accuracy and efficiency of diagnostic processes like polymerase chain reaction (PCR) and chemical synthesis. These small components are pivotal for making POCT devices compact and portable [56]. Microheaters integrated in microfluidic devices will precisely control the temperature of small amounts of fluids or biological samples [57]. They enable controlled heating steps necessary for chemical reactions to proceed efficiently and selectively, ensuring reproducible experimental conditions. They are used in particular for the microfluidic PCR, where the DNA needs to be heated and cooled repeatedly to be properly amplified. Microsensors in POCT devices measure various factors such as temperature, pH levels, chemical concentrations, and interactions between biomolecules [58]. Microsensors offer high sensitivity, capable of detecting picograms of analytes within small sample volumes, enabling the real-time detection and quantification of biomolecules, and reducing the time required for diagnostic processes [59,60] (consult Supplementary Materials—Table S1).

Microfluidics are also used for DNA amplification, sequencing, and analysis, facilitating applications like genetic testing and personalized medicine. Microfluidic devices revolutionize DNA analysis by reducing contamination risks, ensuring swift on-site applicability, and integrating comprehensive analysis workflows. These systems enable advanced techniques like on-chip PCR, digital PCR, and isothermal amplification, enhancing sensitivity and quantification. Commercially available microfluidic techniques demonstrate accessibility and maturity in forensic applications [61].

Microfluidic techniques enable the precise control of fluids and particles at the nanoliter scale, facilitating the simultaneous manipulation and analysis of cultured cells (see Figure 1b) from single cells to intact tissues. The integration of microfluidic devices has advanced quantitative and systems biology, offering a comprehensive approach to studying cellular behavior [62].

Microfluidic devices showcase their broad applicability in environmental monitoring, addressing the challenges in sample introduction and matrix interferences. Despite persistent issues like small sample volumes and low limits of detection, these devices offer enhanced throughput and portability, generating valuable temporal and spatial information. The need for an improved interface between the environment and microfluidic devices is identified. Integrated preconcentration and separation techniques prove effective in overcoming matrix interference challenges. The demonstrated sensitivity of microfluidic devices meets the environmental application requirements, with potential for further advancements through integration with wireless communication technologies [63].

Microfluidic devices offer substantial advantages in quality control. Traditional quality control testing is time-consuming and demands expensive analytical equipment and significant lab space. Microfluidic quality control systems are gaining attention due to their capacity for drastically reducing sample and reagent consumption, shortening analysis times, enhancing detection sensitivity, enabling increased multiplexing, and reducing instrumentation size [64].

Microfluidic devices also offer significant advantages in liquid handling, particularly in revolutionizing technologies that demand precise fluid control, rapid responses, cost-effectiveness, and automation. The field is divided into continuous-flow microfluidics and digital microfluidics, with recent advances in continuous-flow microfluidics focusing on two pivotal applications: mixing and separation. These processes are crucial in LoC platforms for various biological and chemical assays, enhancing sample preparation and detection [65].

In protein analysis, microfluidic devices play a crucial role, driven by the evolving landscape of genomics and proteomics. The importance stems from the distinct requirements of modern biology, necessitating extreme sensitivity, reproducibility, high-throughput capabilities, and the extraction of diverse structural and biochemical information. The shift from macroscale to microanalytical devices is motivated by factors like low sample consumption, rapid analysis times, high-throughput potential, integration and automation possibilities, and the demand for single-use devices to prevent cross-contamination [66].

#### 2.4.2. Organ-on-a-Chip

In biomedical engineering applications, microfluidic systems have been developed to be combined with cells, recreating the human microenvironment, cellular functions, and interactions in several organs. This junction is most known as OoC [67]. OoC can be fabricated in glass, polymers, and plastic to have the advantages already described (like being optically clear) and to enable the cells to mimic the tissue physiology while in vivo [68]. This type of device has a big interest in evaluating the impact and toxicity of new drugs, specific treatments, or therapeutics, given that it is possible to recreate a biochemical human environment, for healthy cases and to recreate several diseases. OoC can be considered a bridging technology when compared to the two-dimensional (2D) in vitro cell culture (that has the problem of failing to recreate the principal physical and biochemical cues), three-dimensional cell cultures, and organoids, offering the ability to work with complex cell cultures and better-designed microenvironments to maximize this type of model [69,70]. The use of OoC devices has also been an important step in reducing the use of animals for testing; numbers since 2011 have been decreasing, and after 2011, are below 10 million. Animal testing also requires large amounts of funding, and they carry the ethical burden of sacrificing animals in experiments.

**Figure 2:**
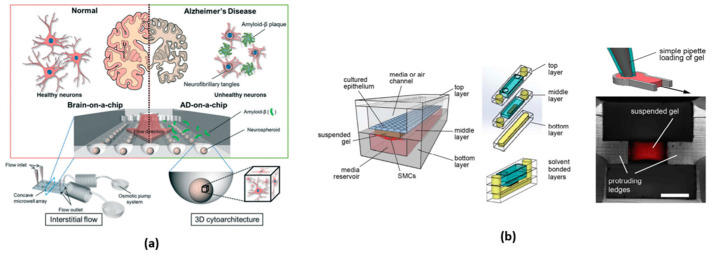
Organ-on-a-chip: (**a**) diagram of a three-dimensional brain-on-a-chip with an interstitial level of flow (obtained from [71]), and (**b**) lung airway-on-a-chip device design and set-up (obtained from [74]).

Nowadays, every individual chip is meticulously crafted to suit its specific purpose, featuring dedicated compartments for accommodating tissues and intricate microchannels that facilitate communication and the seamless flow of fluids among various tissue types. They are fabricated using photolithography and soft lithography or 3D printing. The OoC devices are used to study several organs and disease conditions, such as Alzheimer’s disease (Figure 2a) [71], amyotrophic lateral sclerosis [72], tumor metastasis [73], lung-on-a-chip (Figure 2b) [74], heart-on-a-chip [75], and innumerable other cases [70].

The progression of tumors—including their ability to spread to other parts of the body—is greatly impacted by various elements within the microenvironment surrounding the tumor. As a result, there is a growing effort to develop in vitro 3D models that mimic the tumor microenvironment, enabling researchers to explore how metastasis works and how tumors respond to treatments in a more controlled environment. One such innovative 3D model is known as ToC technology, which offers a potent in vitro platform for cancer research. This technology can replicate the complex physiological structure of tumors and it provides precise control over their spatial and temporal aspects. ToC technology includes features like 3D scaffolding, the cultivation of multiple types of cells, and the simulation of dynamic fluid flow in the human body. Looking ahead, these ToC systems have the potential to evaluate personalized therapeutic approaches for individuals, using samples of their cancer cells obtained through procedures like biopsies. This promises to significantly contribute to the advancement of personalized medicine, thereby improving treatment outcomes for cancer patients [76].

#### 2.4.3. Cell Culture

Cell culture is a critical component of the OoC systems, as it involves the cultivation of human cells within these microfluidic devices to replicate the physiological environment of specific organs. Human cells relevant to the targeted organ are sourced, often from primary tissue samples or cell lines. The cells are seeded onto a substrate within the microfluidic device. This substrate is usually coated with extracellular matrix proteins or other biomaterials to promote cell adhesion and growth. The seeding process must be carefully controlled to ensure the uniform distribution of cells and proper attachment to the substrate. Once seeded, the cells are maintained under controlled conditions that mimic the physiological environment of the organ being modeled. This includes providing the appropriate nutrients, oxygen levels, pH, and mechanical stimuli. Microfluidic channels within the device allow for precise control over these environmental factors. In some cases, the cultured cells may undergo differentiation to better mimic the specialized functions of the target organ. The cell culture component is integrated with the microfluidic system of the OoC device, allowing for the precise control and manipulation of fluid flow, nutrient delivery, and waste removal. This integration is essential for recreating dynamic physiological conditions within the OoC [77].

**Figure 3:**
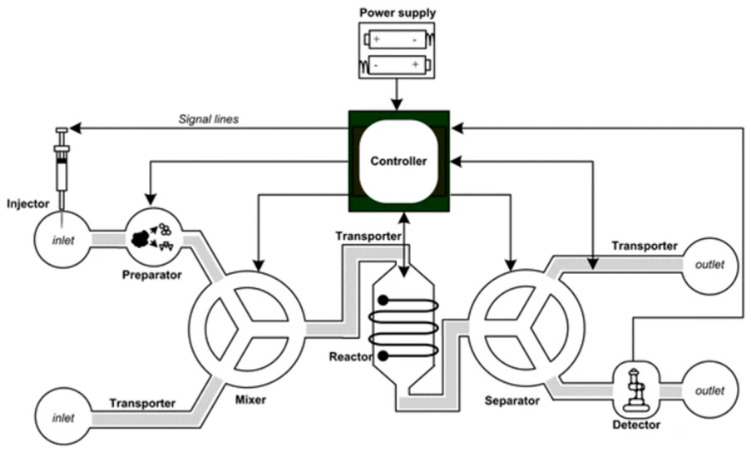
Schematic representation of a lab-on-chip (obtained from [79]).

LoC systems, born from multidisciplinary microfluidic research, boast advantages such as unparalleled speed and cost-effectiveness (see Figure 3). These customizable devices cater to specific applications, integrating fluidics, micromachining, electromagnetics, materials, and chemistry. A revolutionary tool for chemical and biological analyses, they enable self-testing, overcoming spatial constraints. With recent achievements and ongoing innovation, LoC systems offer a compact, efficient, and continuously evolving solution for diverse research needs [78].

#### 2.4.4. Drug Testing

Drug delivery in a microfluidic device is a field that combines principles of microfluidics, drug formulation, and biomedical engineering to develop innovative platforms for controlled and targeted drug delivery. Microfluidic devices offer precise control over the fluid flow, mixing, and reactions, making them ideal for drug delivery applications. Drug delivery in microfluidic devices often involves the precise mixing and encapsulation of drugs with carriers or nanoparticles to enhance their solubility, stability, and targeting capabilities. Microfluidic platforms enable the rapid screening and optimization of drug formulations by varying parameters such as flow rates, concentrations, and mixing ratios [80]. These devices can achieve controlled drug release profiles by integrating various mechanisms such as diffusion, degradation, or stimuli-responsive materials. One of the significant advantages of microfluidic drug delivery systems is their ability to precisely target specific tissues or cells. Functionalizing the surfaces of microfluidic devices with ligands or antibodies allows for the selective binding and delivery of drugs to target cells or receptors, minimizing off-target effects and improving therapeutic efficacy. Microfluidic platforms enable high-throughput screening of drug formulations and delivery strategies due to their small sample volumes and parallel processing capabilities. This accelerates the development and optimization of drug delivery systems, leading to faster translation from bench to bedside [9].

Microfluidic devices can be tailored to individual patient requirements, allowing for personalized drug delivery regimens. By integrating sensing and feedback mechanisms, these devices can dynamically adjust drug doses in real-time based on physiological parameters or biomarker levels, optimizing therapeutic outcomes while minimizing side effects [81]. M. Björnmalm et al., 2014 [81], developed a 3D nanostructured microfluidic chip for the efficient capture of exosomes, nanovesicles linked to lipid membranes, with potential applications in drug delivery therapy. Exosomes arouse great interest due to their therapeutic potential but are limited by their low targeting capacity and the lack of efficient isolation techniques. The presented microfluidic chip utilizes arrays of micropillars functionalized with multi-walled carbon nanotubes to capture exosomes in a highly efficient manner, combining a specific recognition molecule with the unique topography of the nanomaterials. This approach enabled efficient drug delivery to tumor cells, resulting in a significant improvement in the anticancer effect of chemotherapy drugs [81].

#### 2.4.5. Gene Delivery

Gene delivery on a chip, within a microfluidic context, refers to the use of microfluidic devices to deliver genetic material (such as DNA, RNA, or gene-editing tools like CRISPR-Cas9) into target cells or tissues with high precision and efficiency. This approach holds significant promise for various biomedical applications, including gene therapy, genetic engineering, and fundamental research in molecular biology. Microfluidic devices allow for the precise control of fluid flow and manipulation of small volumes of reagents. This miniaturization enables researchers to perform highly controlled and efficient gene delivery experiments with minimal sample and reagent consumption. Researchers can precisely control the location and timing of gene delivery events within the microfluidic channels, enabling spatially resolved studies and dynamic analysis of cellular responses to genetic manipulation. Microfluidic platforms can be designed to perform high-throughput gene delivery screening assays, allowing for the rapid screening of various genetic constructs, delivery methods, and cell types. This capability is particularly valuable for identifying optimal gene delivery strategies for specific applications or therapeutic targets. The capability of recreating complex cellular microenvironments allows researchers to study how the cellular microenvironment influences the efficiency and efficacy of gene delivery and gene expression. The integration of analysis techniques in microfluidic devices enables the real-time monitoring of gene delivery processes, quantification of gene expression levels, and analysis of cellular responses at the single-cell level. Microfluidic platforms are highly versatile and customizable, allowing researchers to tailor the design and functionality of the devices to specific experimental requirements. This flexibility enables the development of innovative gene delivery approaches and the exploration of novel research questions in molecular biology and biomedicine [82].

#### 2.4.6. Microfluidic Cell Sorting

Microfluidic cell sorting is a technique used in biology and biotechnology to separate cells based on various characteristics such as size, shape, electric charge, or biomolecular markers. It utilizes microfluidic devices, which are small-scale systems that manipulate fluids at the microscale level, typically ranging from microliters to picoliters. The basic principle of microfluidic cell sorting involves the controlled flow of cells through microchannels or chambers within the device. Different cells may respond differently to various forces exerted within these channels, allowing for their separation based on specific parameters. Despite their small size, microfluidic devices can process cells at high speeds, enabling a rapid sorting of large cell populations. Microfluidic cell sorting can be easily integrated with automated systems, allowing for high-throughput and reproducible sorting processes. Multiple sorting parameters can be simultaneously assessed and exploited for sorting, enabling more precise and complex sorting strategies [83].

## 3. Numerical Simulation of Microfluidic Devices

Numerical simulation is a computational method that solves mathematical models in order to analyze and study the behavior of complex systems. It involves the creation of algorithms and the application of computer programs to solve equations that describe physical, chemical, and biological processes [84]. In microfluidics, numerical simulation is extremely useful; it allows for testing diverse designs and parameters before the fabrication process, being helpful in predicting the performance of the system. It supports the optimization, efficacy, and functionality of the devices without the necessity of producing multiple prototypes, thereby saving time, resources, and costs [2]. Overall, numerical simulation plays a critical role in accelerating innovation and expanding the application potential of microfluidic devices in biomedical research, diagnostics, and beyond.

### 3.1. Fluid Flow Modeling

Microfluidic simulation delves into the complex dynamics of fluid flow at the microscale, where distinct considerations shape the behavior of fluids. In this realm, a low *Re* (typically below 100) flow prevails, emphasizing the dominance of viscous forces over inertial forces [85]. The equation of the Reynolds number is given by Equation (3) [85]:

(3) $$Re=\frac{\rho vL}{\mu }$$

where ρ is the flow density, v is the fluid velocity, L is the dimension of the system, and *μ* is the fluid kinematic viscosity.

Laminar flow, characterized by smooth layers, is a prominent feature in microchannels, offering precision and controlled fluid manipulation. Laminar flow in microfluidic channels is highly predictable, with well-defined velocity profiles. Due to the absence of significant turbulence, mixing between different fluid streams in laminar flow is limited. This property can be both advantageous and challenging in microfluidic applications. On one hand, it enables precise control over fluid streams and allows for predictable reactions and analyses. On the other hand, it necessitates the use of specialized mixing strategies to achieve efficient mixing when required [86]. In laminar flow, mass transport is primarily driven by diffusion rather than convection. This is because the velocity gradients across the channel are relatively small, resulting in slower convective transport compared to diffusive transport. As a result, mass transport phenomena, such as molecular diffusion and dispersion, play a significant role in microfluidic systems. Laminar flow in microchannels generates low shear stress at the channel walls, which is advantageous for handling delicate biological samples and cells. This characteristic makes microfluidics well-suited for applications such as cell manipulation, tissue engineering, and drug delivery [87].

Fluid movement in microfluidic devices is often pressure-driven, enabling meticulous control of the overflow rates through the application of pressure gradients along microchannels [88]. The scaling laws governing microfluidic flow differ from macroscale systems, accentuating effects such as surface tension and viscous forces [89]. Simulation techniques, notably CFD, facilitate the exploration of flow patterns by incorporating microchannel geometry, fluid properties, and boundary conditions [90]. The increased flow resistance and capillary effects in microchannels, coupled with surface interactions like wetting, play pivotal roles in dictating flow behavior [91]. Efficient mixing and dispersion, essential for diverse applications, depend on a comprehensive understanding of fluid-flow patterns within microfluidic systems [92]. Temperature effects also come into play, necessitating the consideration of thermal variations due to the small volumes and high surface-area-to-volume ratios inherent in microfluidics [93].

The Navier–Stokes equations (Equation (1)) describe the motion of viscous fluids and they are fundamental for modeling fluid flow in microfluidic systems. They consist of the continuity equation and the momentum equations, which account for mass conservation and the balance of forces acting on the fluid, respectively [94].

In addition to fluid flow, microfluidic simulations often involve the transport of solutes or particles within the fluid. The convection–diffusion equation describes the combined effects of advection (fluid flow) and diffusion on the distribution of solutes or particles within the fluid [95]. The momentum conservation is presented in Equation (4) [95]:

(4) $$\nabla \vec{v}=0$$

Convection–diffusion is described by Equation (5) [95]:

(5) $$\frac{\partial c}{\partial t}+\nabla \left(cv\right)=D\nabla^{2}c$$

where c is the concentration of the solute or particles, D is the diffusion coefficient, t is the time, and v is the velocity vector.

The Grashof number (*Gr*) is a dimensionless number used in fluid mechanics and in heat transfer to characterize the importance of the buoyancy force relative to the viscous force in a fluid. This number is given by Equation (6) [96]:

(6) $$Gr=\frac{g\beta \Delta TL^{3}}{v^{2}}$$

where g is the gravitational acceleration, β is the thermal expansion coefficient of the fluid, ΔT is the temperature difference between the hot and cold surfaces, and v is the kinematic viscosity of the fluid.

In essence, microfluidic simulations serve as indispensable tools for designing and optimizing devices, providing insights into the nuanced fluid-flow phenomena at the microscale. These simulations contribute to advancements in medical diagnostics, chemical analysis, and other fields by unraveling the complexities of microfluidic flow [97].

### 3.2. Microfluidic Devices’ Design Optimizations 

**Figure 4:**
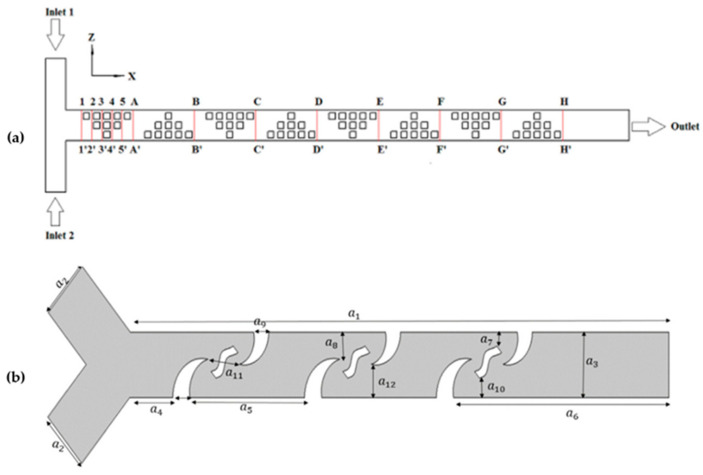
(**a**) Diagram of cross-sections of T-type micromixer (obtained from [98]). (**b**) Proposed micromixer with baffles design to improve advection-based mixing (2D view) (obtained from [100]).

Chen et al., 2017 [98], aimed to optimize the design of obstacles in a three-dimensional T-type micromixer (see Figure 4a). They conducted a numerical analysis to investigate how the direction of flow velocity constantly changes due to the blocking obstacles, which generates chaotic convection and effectively increases species mixing. Additionally, they applied the orthogonal experiment method to determine the effects of some key parameters on mixing efficiency. The results show that the height of obstacles has the greatest weight in mixing efficiency, followed by geometric shape, symmetry, and number of obstacles. Based on the optimized results, the researchers designed a multi-unit obstacle micromixer. This new design is compared with the T-type micromixer, demonstrating that the multi-unit obstacle micromixer is more efficient, achieving over 90% mixing efficiency for a wide range of Peclet numbers. The results suggest that the optimal design method of obstacle layout in three-dimensional microchannels is a simple and effective technology to improve species mixing in microfluidic devices. Additionally, the authors highlight the potential of this methodology for applications in chemical engineering and bioengineering. This research contributes to the development of advanced mixing techniques at the microscale, with significant implications in various areas, including chemical analysis, medical diagnostics, and microfluidic device fabrication [98].

Chen et al., 2016 [99], focused on optimizing the shape of microchannels in passive micromixers. They aimed to demonstrate that altering the shape of microchannels is a straightforward approach to enhancing species’ mixing efficiency in passive micromixers. Through numerical analysis, they observed that the constant change in flow velocity direction, caused by the microchannels’ shape variations, leads to chaotic convection, effectively improving species mixing. The researchers conducted simulations and analyses involving six different channel shapes. They validated their findings through multiple mixing experiments and further analyzed the advantages of square-wave micromixers. Their simulations indicated that square-wave microchannels outperformed other shapes in enhancing species mixing. Additionally, they found that by adjusting the number of square-wave units, even better mixing performance could be achieved. The study concludes that shape optimization of microchannels represents a simple, flexible, and efficient method for improving micromixer mixing performance. They assert that this design approach has the potential to significantly enhance species mixing in microfluidic devices. Moreover, they suggest that the shape optimization methodology could find applications in the system-level design of lab-on-a-chip devices [99]. Dehghani et al., 2020 [100], focused on utilizing microfluidics for efficient and low-reagent-consumption experiments, with micromixers being a crucial component of integrated microfluidic systems (see Figure 4b). They investigated enhancing the mixing of microfluids using a passive method, specifically by introducing new types of baffles designed through topology optimization to improve advection-based mixing. The research employed numerical simulations conducted with COMSOL Multiphysics software. The simulation results were validated using experimental data obtained from a manufactured sample of the designed micromixer. The designed geometry was found to facilitate rapid mixing within a short distance along the microchannel. The study observed that as the Reynolds number increased from Re = 0.2 to 5, the quality of mixing decreased, indicating a transition to a pure diffusion-based mixing mechanism. Consequently, higher Reynolds numbers resulted in a lower residence time and mixing index. However, beyond Re = 5, increasing the Reynolds number led to an improvement in the mixing index, reaching MI (mixing index) = 0.98 at Re = 100. The formation of vortices behind the designed baffles at Reynolds numbers exceeding 5 served as evidence for enhanced mixing. Overall, the research demonstrates the effectiveness of the proposed passive micromixer design in enhancing microfluidic mixing, particularly at higher Reynolds numbers, where advection-based mechanisms dominate [100].

### 3.3. Fluid Flow Analyses

To effectively understand and optimize the microflow within channels and track the internal microfluidic parameters, it is essential to first visualize the microscale flow in simulation and then validate it through experimental visualization, both conducted at the same scale. Researchers in fluid mechanics have been working on visualizing fluid-flow patterns since the early 2000s. They aim to achieve high spatial resolution using relatively simple optical methods [101].

The ANSYS Fluent software and the COMSOL are particularly effective tools for studying and analyzing microfluidic flow behaviors. This computational analysis method has several advantages, including robust device design and the ability to quickly simulate complex and coupled physics in a low-cost way [102].

**Figure 5:**
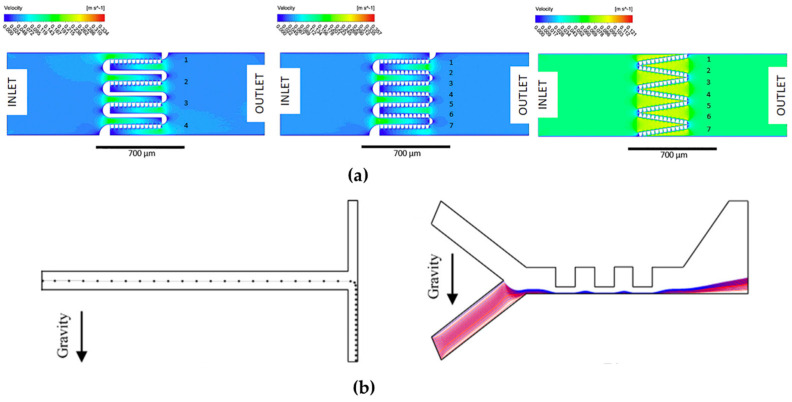
(**a**) Velocity plots of the three investigated geometries for efficient blood–plasma separation (adapted from [103]). (**b**) The numerical model results for size-based separation of polystyrene microparticles (ρ water = 995.09 kg/m^3^ and ρ particles = 1060 kg/m^3^) (obtained from [104]).

The Ansys Fluent has been used in several studies. Spigarelli et al., 2019 [103], described the challenge of blood plasma separation in point-of-care devices to retain specific cells or analyze plasma biomarkers. The researchers developed a novel passive device that employs a size-exclusion method for this purpose. The research process began with the design of a filter resembling a particulate trap, and numerical simulations using ANSYS Fluent were conducted to optimize its geometry for efficient blood–plasma separation (see Figure 5a). The finalized filter design was then incorporated into a microfluidic channel, and a master prototype was created using standard cleanroom manufacturing processes. To replicate the device on a larger scale, a 1 mm thick cyclic olefin copolymer wafer was selected for hot-embossing replication. This choice was made because cyclic olefin copolymer is transparent, resistant to solvents, and meets medical-grade standards. Additionally, the chosen manufacturing process can be easily scaled up for industrial production [103].

Sanjuan et al., 2022 [104], investigated the thermo diffusion phenomenon both through numerical simulations and experimental testing to achieve a size-based separation of polystyrene microparticles. To validate their model, they used ANSYS Fluent software (see Figure 5b). For their experimental analysis, they designed a novel microchannel configuration intended to separate two distinct groups of particles measuring 5 and 20 μm in size. The microparticles’ trajectories were examined within the central channel of the microdevice under various conditions: without a temperature gradient, with a thermal gradient applied parallel to gravity (cooling from either the bottom or top), and with a temperature gradient perpendicular to the direction of the gravitational force. The combined numerical and experimental findings for these specific geometries and boundary conditions led to the conclusion that, under typical terrestrial conditions, microparticles measuring 5 μm and larger made of polystyrene cannot be effectively separated through thermophoresis inflow due to the influence of gravity [104].

Jeong et al., 2021 [105], addressed the challenge of developing therapeutics for neurological diseases, where most compounds cannot pass through the blood–brain barrier. The study investigated the effects of various factors, such as volumetric flow rates, polycarbonate membrane porosities in the blood–brain barrier model, and microfluidic channel dimensions, on shear stress in vivo. The results showed that decreasing the microfluidic channel dimension and reducing porosity increased shear stress in vivo. The simulation predictions based on the numerical approach closely matched the experimental values of the in vivo shear stress, with an error of only 2.17% at a porosity of 0.01% and all volume flow rates. This method offers a standard for developing organ-on-a-chip microfluidic devices that replicate in vivo conditions and shear stress accurately. This advance is crucial to improving drug delivery and studying neurological diseases more effectively [105].

**Figure 6:**
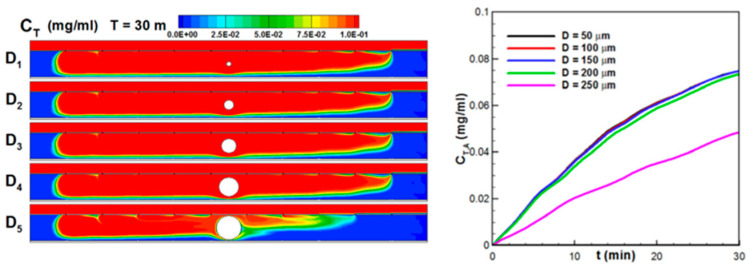
Average drug concentration distribution in the tumor channel with five different tumor diameters (obtained from [106]).

Li et al., 2019 [106], focused on addressing the challenge of effectively delivering anticancer drugs to tumor sites, where precise drug transport and distribution within the tumor microenvironment is crucial for therapeutic success. The research employed a combination of numerical modeling using Ansys Fluent and a microfluidic platform called the tumor-vasculature-on-a-chip model to investigate the dynamics of drug transport (see Figure 6). The main objective of the study was to understand how different factors, such as tumor size, number of tumors, and their positioning, affect the distribution of anticancer drugs within the tumor microenvironment. The numerical simulation was initially validated using experimental data, ensuring that the computational model accurately represented real-world drug transport dynamics. This research established a connection between the size and positioning of solid tumors and the uneven distribution of drugs in the tumor microenvironment. Understanding this relationship can help researchers and clinicians improve anticancer drug delivery to ensure more effective treatments for patients, particularly those with heterogeneous drug distribution in their tumors [106].

### 3.4. Microfluidic Mass Transport Phenomena

Mass transport within microfluidic simulations encompasses the intricate movement and dispersion of solutes or particles in minute fluid channels or devices [107]. At the microscale, diffusion takes precedence due to the diminutive dimensions involved, acting as the primary mechanism driving molecules or particles from regions of higher concentration to those of lower concentration [2]. Concurrently, controlled fluid flow, propelled by pressure or similar mechanisms, introduces further, influencing the efficient transport of solutes [108].

Efficient mixing, a pivotal aspect in microfluidic devices, capitalizes on the interplay of diffusion, convection, and advection. The precisely designed flow patterns and reduced characteristic lengths in microchannels facilitate rapid and accurate mixing, catering to diverse applications [92]. Taylor dispersion, observed in microfluidic channels, contributes to the axial dispersion of solutes, resulting from the parabolic velocity profile of the fluid. This phenomenon leads to the broadening of concentration profiles along the length of the channel [109].

Interactions at fluid–solid or fluid–liquid interfaces significantly affect mass transport within microfluidic systems. Surface characteristics, such as wettability, exert an influence on the transfer of solutes, impacting their behavior within the microchannel [110]. Microfluidic simulations must account for the distinct properties of different solutes or particles, including diffusivity, size, and chemical interactions [111]. Utilizing computational models like finite element (FE) and CFD, researchers simulate mass transport by considering microchannel geometry, fluid properties, and boundary conditions. These simulations prove invaluable in predicting and optimizing the distribution of solutes over time, contributing to the design of efficient microfluidic devices for applications such as chemical analysis, drug delivery, and LoC systems [112,113].

Fick’s first law describes the diffusion of solutes in a fluid medium. It states that the flux of a solute through a unit area perpendicular to the direction of diffusion is proportional to the concentration gradient (Equation (7)) [114]:

(7) ∇J=−∇(Dc),

where J is the solute flux (amount of solute crossing a unit area per unit time), and D is the diffusion coefficient of the solute. Fick’s second law extends Fick’s first law to describe how the concentration of a solute changes with time due to diffusion. Equation (8) describes how the concentration profile of a solute evolves in response to diffusion [114].

(8) $$\frac{\partial C}{\partial t}=D\;\nabla^{2}C,$$

where ∂C/∂t is the rate of temporal variation of concentration C at time t.

### 3.5. Microfluidic Mixing Phenomena

Mixing in microfluidic systems has the principal objective of reaching a rapid and microlevel mixing of all molecules and samples (drug, metabolism products, nutrients, medium of cell culture, among others) present in this type of device. This process is essential and is achieved by making the diffusion and stirring between all the samples in the flow easier, making the distribution uniform, and recreating the biological environment [115,116]. Mixing in microfluidics refers to the process of homogenizing multiple fluid streams or enhancing the interaction between different components within microscale channels. Efficient mixing is crucial for various applications in microfluidics, including chemical synthesis, biochemical assays, sample preparation, and drug discovery. The challenges of mixing in microfluidics arise primarily due to the laminar flow regime and the absence of turbulence. Several mechanisms and strategies are employed to achieve efficient mixing in microfluidic systems. In laminar flow, diffusion plays a significant role in mixing. It enables the gradual intermingling of different species by molecular motion. The rate of diffusion is described by Fick’s laws of diffusion. In contrast, laminar flow limits convective mixing, and controlled advection can be utilized to enhance mixing. This can be achieved through various methods, such as inducing secondary flows (Dean flow), utilizing microfluidic geometries, or applying external forces [117]. Many factors influence the mixing in microfluidic devices, such as the following:

- The flow velocity: Faster flows lead to little interaction between components, and slower flows lead to an uneven distribution of components [118]. The influence of a laminar flow in a microchannel demonstrates that the diffusive mass flow between two miscible streams flowing in a laminar fashion is increased when the velocity at their diffusion interface is increased [119]. The conditions of the flow can be a continuous or pulsatile fluid flow [120].
- The device materials: The use of hydrophobic materials can lead to the accumulation of fluid in certain areas or bubble formation, which can be improved by using a superficial modification [46].
- The mixing techniques: In general terms, microfluidic mixing methods can be classified into two main categories: “active”, where an external energy force is used to disrupt the sample species, or “passive”, where specially designed microchannel configurations are employed to increase the contact area and contact time of the sample species. Some examples are diffusion, rotation, vibration, pressure gradient, and micromixers [115].
- The size of particles and molecules: Different sizes of particles have different velocities [118].
- Capillarity: Capillarity involves the ability of a liquid to rise/move through narrow spaces due to the attraction between the fluid molecules and the surfaces of the materials that make up these spaces. It occurs due to surface tension and the adhesion and cohesion forces of the liquid molecules [121].
- The geometry: The geometry of a microfluidic device impacts the mixing in several ways. It influences the predominance of convection versus diffusion, determines the residence time of substances, controls the transition between laminar and turbulent flow, creates obstacles and structures that favor mixing, and allows scalability. Furthermore, the introduction of reagents and the specific geometry design can be adjusted for specific applications [92].
- Diffusion: Diffusion plays a key role in mixing in microfluidic devices. It allows the natural homogenization of different components, spreading from areas of higher to lower concentration. This process can be precisely controlled by adjusting the channel width, length, and concentration gradients. Diffusion is particularly effective in laminar flows, reducing the need for turbulent structures and providing controlled, predictable mixing. Its gentle nature minimizes sample loss and dilution, making it ideal for applications where sample integrity is paramount [122].

### 3.6. Organ-on-a-Chip Modeling Studies

An emerging field that has attracted significant attention is OoC numerical simulation. This innovative approach seeks to reproduce the physiological and functional characteristics of human organs in microfluidic devices. In this section of the literature review, the current scenario of numerical simulation applied to the OoC was deepened. Here, light was shed on recent innovations, the challenges faced by the scientific community, and the potential contributions to understanding cellular interactions, responses to specific medications, and the complexities of various pathologies [123].

#### 3.6.1. Lung-on-a-Chip

**Figure 7:**
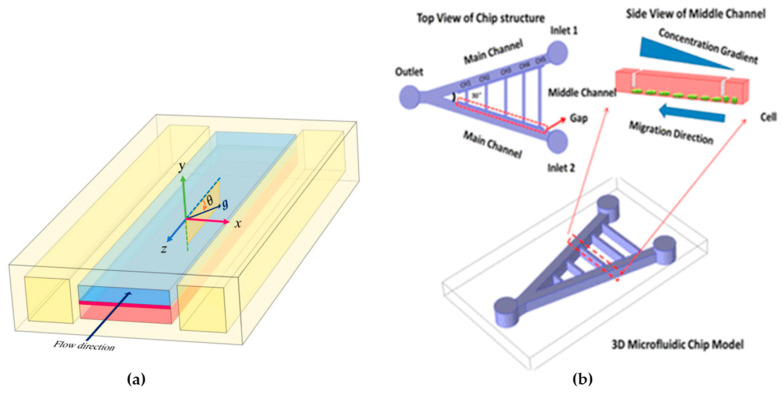
(**a**) Schematic representation of the computational domain, modeled after the LOAC design (obtained from [124]). (**b**) A V-shape double-channel microfluidic device for chemotaxis. Inlet 1 was designed for media flow, and inlet 2 for chemokine perfusion (obtained from [76]).

Amin Arefi et al., 2020 [124], conducted FE simulations to assess the deposition of airborne nanoparticles on the epithelial layer of a lung-on-a-chip (LOAC) device, a model for studying the impact of nanoparticles on human health (see Figure 7a). They addressed the issue of low reproducibility in NP testing by solving Navier–Stokes equations and employing Eulerian advection-diffusion and Lagrangian particle tracking for fine and coarse nanoparticles, respectively. The authors explored nanoparticle adsorption under various conditions, including exercise and breath-holding patterns, simulating physical activity, and smoking scenarios. The researchers identified factors influencing particle deposition, such as air-flow volume, breathing frequency, Brownian motion, and gravitational settling, and the compared simulation results with experimental data reveal qualitative and semi-quantitative agreement. They concluded that computer simulations could enhance the design and application of LOAC devices for analyzing interactions between particles and alveolar tissues [124].

Zou et al., 2015 [125], developed a microfluidic device enabling the creation of multiple stable gradients, on-chip cell culture, and real-time monitoring of single-cell migratory behavior (see Figure 7b). They applied this platform to investigate gradient-induced chemotaxis in lung cancer stem cells (LCSC) and differentiated LCSC (dLCSC). The study revealed dynamic and distinct responses of LCSC and dLCSC to chemotaxis, which is governed by the β-catenin-dependent Wnt signaling pathway. Significantly, the microfluidic analysis demonstrated that LCSC and dLCSC, originating from the same source, exhibited diverse behaviors in response to the same external stimuli, emphasizing the significance of cancer cell heterogeneity. Additionally, the study observed a previously unreported acceleration of both LCSC and dLCSC during chemotaxis, influenced by increasing local concentration in different gradients, a phenomenon uniquely captured through the microfluidic approach. The ability to analyze single-cell chemotaxis under spatially controlled conditions offers a novel analytical platform for exploring cellular microenvironments and understanding cancer cell metastasis [76,125].

#### 3.6.2. Brain-on-a-Chip

Drapaca, 2018 [126], aims to establish the scientific groundwork for creating an implantable brain-on-a-chip, a specialized device designed to monitor and support essential brain functions. The focus is on proposing the structure of this brain-on-a-chip and developing a mathematical model for the combined mechano-electrochemical processes within a neuron and its membrane. The model involves a constrained Lagrangian formulation that connects an electronic membrane model with the motion and diffusion processes in a triphasic porous medium inside the neuron. The triphasic medium consists of solid, fluid, and ionic phases. The paper concludes by presenting a simplified Lagrangian formulation suitable for practical applications [126].

#### 3.6.3. Liver-on-a-Chip

Docci et al., 2022 [127], explored the application of the PhysioMimix liver-on-a-chip system, a microphysiological system designed to mimic human liver functions, for studying multiple drug metabolism applications. The authors characterized the activities of key enzymes—cytochrome P450 (CYP), UDP-glucuronosyl transferase, and aldehyde oxidase—using 15 drugs and assessed an in vitro to in vivo extrapolation for 12 of them. They also investigated the liver-on-a-chip’s utility in estimating the fraction metabolized through specific biotransformation pathways for quinidine and diclofenac. They explored metabolite identification employing mathematical modeling to improve the parameter calculation in non-homogeneous drug concentration scenarios, considering factors like medium flow and evaporation. They highlighted that evaporation can lead to up to a 40% underestimation of intrinsic clearance for low-clearance compounds if not considered. The authors demonstrated the integration of mathematical modeling with liver-on-a-chip studies, enhancing the estimation of the fraction metabolized and supporting the assessment of metabolism models of varying complexity. The study underscores the importance of this integrated approach for generating high-quality data from complex in vitro cellular systems [127].

#### 3.6.4. Multi-Organ-on-a-Chip

**Figure 8:**
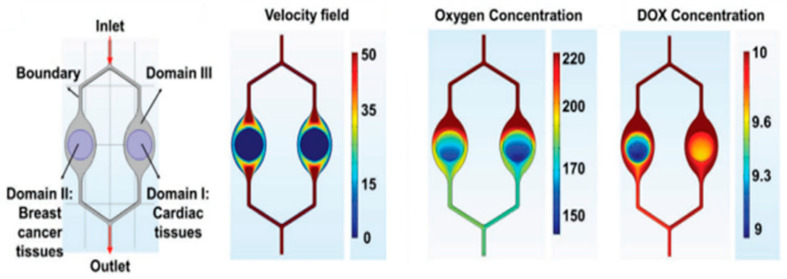
Cardiotoxicity-on-a-chip to non-invasively monitor multiple biomarkers secreted by the cells with aptamers-functionalized biosensors (obtained from [128]).

Lee et al., 2020 [128], proposed a geometry to study cardiotoxicity, which is one of the most concerning side effects of cancer chemotherapy. In the numerical simulations, they model the distribution of the flow rate, oxygen level, and drug concentration (see Figure 8). This “cardiotoxicity-on-a-chip” (CIC) contains heart tissue of induced pluripotent stem cells’ communication with breast cancer tissues and multiplexed electrochemical immuno-aptasensors to non-invasively monitor multiple biomarkers secreted by the cells. The production of multiple biomarkers, as evaluated by electrochemical immuno-aptasensors, closely matched the outcomes obtained through an enzyme-linked immunosorbent assay. This alignment underscores the impressive precision and heightened sensitivity of the detection platform, which boasts significantly lower detection limits for monitoring both CIC and the advancement of breast cancer. Furthermore, the versatility of this platform was confirmed through the comparison of biomarker production rates in both cardiac and breast cancer tissues. They recognize that the scaling of tissue-on-chip was not a consideration in the current study, as its primary objective was to establish a proof of concept for the early detection of CIC symptoms. To further enhance and validate the existing model, it becomes imperative to incorporate vital liver components, which play a role in human drug metabolism, as well as to integrate their sensors within the “chip”. Such advancements in the functionality of this platform hold the potential to enable the early detection and prediction of CIC in individual patients in the future [128].

**Figure 9:**
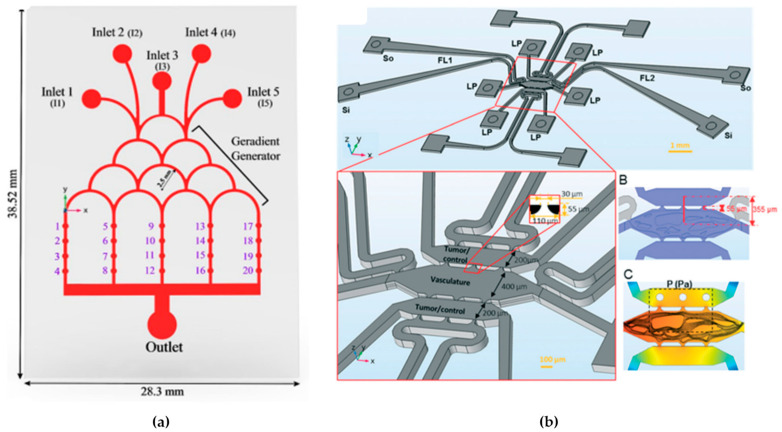
(**a**) Top-view of the first ToC design consisting of twenty microwells (four microwells per channel) and the inlets and outlets for drugs and cells (obtained from [129]). (**b**) The convection–diffusion model for tumor-vascular communication within the device comprises three parallel tissue chambers, each connected to two square tissue-loading ports (obtained from [130]).

Hajari et al., 2021 [129], studied the performance of microfluidic devices, such as the ToC, that enable the delivery of multiple drugs for diverse treatments, such as cancer treatment. They conducted numerical simulations to evaluate the performance of the ToC, focusing on combining multiple nanodrugs and optimizing the device. The simulations considered the typical chip design. The researchers investigated the impact of various parameters, such as cell injection time and location, inlet flow rate, medium viscosity, and cell concentration, on the chip’s performance in terms of shear stress and cell distribution. The results highlighted the importance of operational parameters to prevent the ejection of spheroids from microcavities and maintain shear stresses in a physiological range. Furthermore, they showed that using three inputs on the chip improves uniformity in cell distribution compared to one or two inputs. Based on the results of the simulations, the researchers optimized the ToC design, allowing the simultaneous application of multiple nanodrugs according to each patient’s needs. This optimization also ensured the uniform distribution of cells to generate uniformly sized tumor spheroids and perform complete medium exchanges (see Figure 9a). It has been determined that factors such as cell injection location and strategy, average viscosity, inlet flow rate, and cell concentration have a significant influence on cell distribution within the ToC. Moreover, the precise adjustment of the inlet flow rate for each drug allows for immediate modification and administration of different combinations of nano drugs as desired. In summary, this study demonstrates the effectiveness of numerical simulations in visualizing the performance of ToCs and develops a new optimized design for nanodrug-based therapies [129].

Shirure et al., 2018 [130], introduced a microfluidic system that replicates the natural movement of molecules near the arterial end of a capillary within the tumor environment. This system enables the physiological delivery of nutrients and/or drugs to the tumor through the vascular network (see Figure 9b). They have shown that it is possible to cultivate, grow, and treat tumor cell lines and patient-derived breast cancer organoids on this platform. What is particularly noteworthy is that this platform allows them to observe the essential aspects of tumor progression, such as cell proliferation, angiogenesis, cell migration, and the entry of tumor cells into the bloodstream, both simultaneously and dynamically. Additionally, they have confirmed the practicality of this platform for drug discovery and personalized medicine by examining the responses to chemotherapy and antiangiogenic treatments. The effectiveness of precision medicine-based cancer therapies can only be achieved if individual tumors can be promptly evaluated for their sensitivity to treatment within a clinically relevant time frame (within approximately 14 days) [130].

## 4. Geometries for Microfluidic Devices

The geometry has a fundamental importance in the microfluidic devices area. This parameter allows controlling the flow in the device, efficient mixing, reaction conditions (the size and format in reaction cameras), the transport of particles, energetic efficiency, reduction of reagents and samples (geometry to reduce the waste of reagents), and finally the function integration (different functions in one device) [131,132]. This part of the present review paper is an overview of some geometries that allow the improvement of capillarity and mixing in microfluidic devices.

### 4.1. Geometries to Improve the Mixing

To overcome the biggest problems in mixing, various methods of mixing enhancement within microfluidic devices are being explored.

#### 4.1.1. Passive Mixing Methods

These elements are normally incorporated into the microfluidic devices during manufacturing. It is recommended when looking for an easy way to incorporate mixing enhancement in processes that the level of mixing is not a crucial point [19].

**Figure 10:**
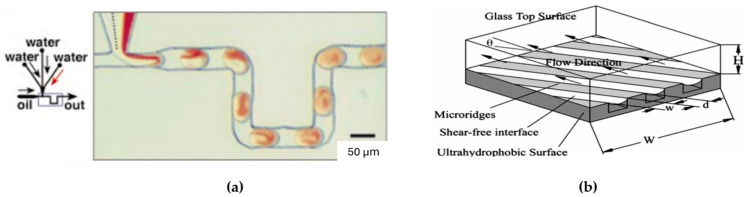
(**a**) A microfluidic device and a microphotograph of plugs (obtained from [136]). (**b**) Schematic diagram of a model for ultra-hydrophobic drag reduction. A combination of surface hydrophobicity and roughness allows water to stand away from the solid surface (obtained from [136]).

- Diffusion and stream splitting/combination: The basis for this method is that two different fluids, when in contact, will mix through molecular diffusion; the mixture between both leads to a uniform mixture at the molecular level. However, in microfluidic devices, this process is typically very slow [133].
- Slanted wells, ridges, or grooves: This method is normally easy to fabricate within a microfluidic device that can provide additional mixing without the need to increase the channel length. The researchers in this area sought to enhance the rate of mixing beyond what could be achieved through diffusion alone. They aimed to achieve this by amplifying the lateral transport within the channel, with the theoretical expectation of reducing the mixing distance. Johnson et al., 2020 [134], developed a device by using an excimer laser system to create slated wells along a fabricated microchannel [134].
- Multiphase micromixing: Simply, the reagents cannot infiltrate the surrounding fluid because of immiscibility, leading them to retain a concentrated state within their droplets (see Figure 10a). This concentration, coupled with other naturally occurring enhancements like chaotic advection or recirculation, has the potential to significantly improve mixing within the droplets [135].
- Hydrophobic surfaces and other passive mixing enhancements: Ou et al., 2004 and 2007 [136,137], investigated the application of hydrophobic surfaces with micro ridges to enhance mixing in microfluidic systems (see Figure 10b). The hydrophobic surface introduces a shear-free air–liquid interface by increasing the contact angle between the fluid and the channel walls. This, in turn, leads to a reduction in drag forces on the fluid by over 40% [136,137].

#### 4.1.2. Active Mixing Methods

These methods offer superior control over the level of mixing obtained, and with this, they are more complex and have a more expensive fabrication [19]. Some are presented here.

**Figure 11:**
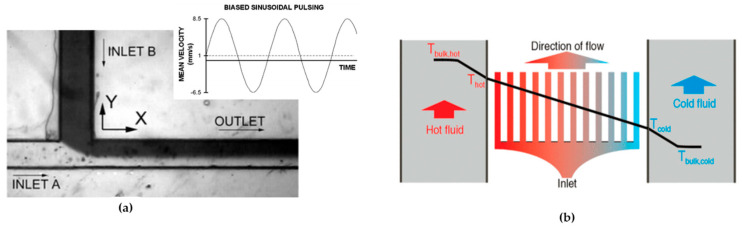
(**a**) Microscope image showing two inlet channels, in line and perpendicular to the outlet channel; and a graph representation of inlet means velocity as a function of time in the control case (dashed line) and in the biased sinusoidal pulsing case (solid line) (obtained from [140]). (**b**) Illustration of the concept of a linear temperature gradient microfluidic system (obtained from [141]).

- Microstirrers: These can be used in microfluidic devices to improve mixing, similar to how a magnetic stirring bar is used in larger-scale mixing processes. This method involves a rotating magnetic field that induces the rotation of a microbar within a fluid system, thereby enhancing mixing in the immediate vicinity of the bar. To further improve mixing, multiple bars can be used simultaneously [138].
- Acoustic mixing: Frommelt et al., 2008 [139], researched employing surface acoustic waves to create time-dependent flow patterns aimed at enhancing mixing in microfluidic devices. These surface acoustic waves represent a form of elastic energy propagating along the fluid’s surface, capable of inducing acoustic streaming within the fluid upon excitation. The resulting streamlines have demonstrated the ability to facilitate effective mixing in fluid systems and the manipulation of liquid droplets, even under conditions of low Reynolds numbers [139].
- Periodic fluid pulsation: This approach introduces a ‘pulsed’ flow in one or both channels to enhance mixing at the interface between the two streams. The pulsation is easily achieved by dynamically altering the voltage applied across the channels, particularly when electroosmotic pumping is employed. Because the flow is not constant, the streamlines undergo constant changes, leading to induced alterations in the flow patterns of the fluids within the system (see Figure 11a) [140].
- Thermal mixing enhancement: The usual trend is for diffusion coefficients to rise as the temperature increases. Mao et al., 2002 [141], showcased the creation of linear temperature gradients in a microfluidic system that spans multiple parallel streams simultaneously. While these gradients may not inherently improve mixing in microfluidic devices, the capability to accurately regulate the fluid’s temperature can empower users to manage the diffusion rate within the streams more effectively (see Figure 11b) [141].

### 4.2. Geometries with Improved Capillarity

The exceptional hydrophilicity of channel surfaces represents a pivotal concern in the context of microfluidic systems, as it enables the efficient manipulation of liquids without the necessity of additional pumping mechanisms. To enhance hydrophilic performance, the customary approach involves surface modifications aimed at transforming the inherent hydrophobic characteristics of PDMS or SU-8 microfluidic channels. Nonetheless, a recurring issue emerges with PDMS surfaces, characterized by hydrophobic rebound when traditional plasma methods are employed for short-term hydrophilicity adjustments. As a result, for the creation of enduring microfluidic chips, the adoption of naturally hydrophilic glass serves as a viable alternative, albeit requiring a more intricate and time-intensive manufacturing process. When expediting the production of microfluidic chips is a priority, laser micromachining stands as an attractive option due to its simplified, rapid, and direct writing capabilities, accommodating a diverse range of geometric shapes far more efficiently than conventional photolithography and etching techniques. Consequently, employing CO2 laser ablation and/or polymer molding becomes a feasible approach for crafting fluidic microstructures in PMMA or PDMS, suitable for applications in micromixers, capillary pumping, and bio-MEMS. An established integration process involves the utilization of laser-ablated PMMA molds, followed by PDMS casting and surface modifications, which can be adapted for a broad spectrum of microstructures and diverse application scenarios. Addressing the hydrophobic nature of PDMS surfaces emerges as another crucial aspect for enhancing mixing efficiency. Furthermore, the fabrication of dual-tone convex-and-concave PDMS microstructures for microfluidic chips introduces an additional layer of complexity and challenge [142].

## 5. Conclusions and Future Perspectives

Microfluidic devices and the simulation of these systems have revolutionized the biomedical engineering area, which includes microfluidic systems dedicated to diagnosis applications, point-of-care devices, drug delivery, cell culture in OoC devices, biosensors, and wearable and implantable devices. Microfluidic systems usually offer better performance in analysis, have the ability to combine many functions, are easier to automate and control, use fewer substances, are safer, cost less, are more sensitive, and provide faster results compared to larger conventional systems.

The ability to model and predict the behavior of fluids at the microscale, within channels and devices of micrometric dimensions, has profound implications in various fields of science and technology, making numerical modeling a constantly growing field. This growth gives valuable insights to create new designs and optimizations of microfluidic systems.

After all, numerical simulation still has limitations and challenges since biomedical devices are becoming more and more complex. Taking that into account, it is important that numerical simulation of these types of platforms becomes more accurate, something that takes time since the chemical and physical processes occur at a micro and macro level, making the numerical simulation difficult. Even knowing these limitations, it is expected that this field will grow and become more realistic to better replicate what happens in vitro and in vivo. Hence, it is expected that numerical modeling will promote the acceleration and progression of science and technology in biomicrofluidics, and innovation in diagnosis, biotechnology, and LoC areas.

With advancements in simulation technology and computational hardware, it is expected that the resolution and accuracy of simulations will increase. This will enable more accurate modeling of complex phenomena in microchannels and microdevices. Future simulations are likely to integrate multiple physical phenomena such as mass transport, heat transfer, electrochemistry, chemical reactions, and fluid–structure interactions. This will provide a more holistic and precise understanding of the behavior of biomicrofluidic systems. As the understanding of biomicrofluidic phenomena progresses, the development of new mathematical models that more accurately capture the details of physical processes at the microscale is anticipated. This may involve multi-scale models and hybrid approaches. Numerical simulation can play a fundamental role in optimizing microfluidic devices and associated processes, facilitating more efficient and refined design of microchannels, microreactors, and other devices. These advancements hold the potential to drive significant innovations across various scientific and technological domains and, importantly, support faster clinical translation and commercialization.

Despite the advantages of microfluidics-based devices, they still present challenges and limitations. The versatility of microfluidic technology requires precise control and high precision in the design and fabrication, which are both challenging and costly, thereby limiting large-scale production. The required manufacturing materials must meet the requirements for biocompatibility, chemical resistance, and mechanical stability, further increasing the costs. 

Additionally, achieving precise control of fluid flow in microchannels remains a challenge since a small variation can significantly impact the device’s performance. The maintenance and prevention of failure are difficult, considering their size. Issues like biomolecules’ absorption also affect the accuracy of analytical measurements. All these hitches, along with the strict processes of regulatory approval, can be expensive and time-consuming, creating further difficulties for the commercialization of such devices. Moreover, market acceptance of microfluidic devices can be challenging since there is no substantial proof of cost-benefit outside the research settings.

However, these challenges, ongoing research, and advancements continue to address these limitations. Innovations in materials, manufacturing techniques, and integration strategies are gradually overcoming barriers, paving the way for broader commercialization of microfluidics-based devices in biomedical research, diagnostics, and other applications.

## Figures and Tables

**Table 1:** Commonly used 3D printing techniques to produce microfluidic devices, their advantages, and disadvantages.

Techniques	Advantages	Disadvantages
SLA	High precision and detail Simplified process Variety of resins Automation and medium-scale production Ease of design	Cost of resins Production scale limitations Mechanical properties of resins Material toxicity
FDM	Integration of various materials Lower cost Simplified assembly Versatility and accessibility	Lower resolution Surface roughness Adhesion and sealing Low material resistance (temperature and mechanical stress)
SLM	High precision and detail Rapid prototyping Material versatility Flexibility and cost-effectiveness	Accuracy and surface roughness Equipment cost Material limitation

**Table 2:** Commonly used materials to produce microfluidic devices, their advantages, and disadvantages.

Materials	Advantages	Disadvantages
Glass [45]	Transparency; Chemical compatibility; Dimensional stability; Withstands high temperatures; Biocompatibility.	High cost; Fragile; Difficult to fabricate; Does not support the integration of electronic components.
Silicon [15]	Flexibility and elasticity; Biocompatibility; Easy to fabricate; Chemical resistance; Optical transparency.	Gas permeability; Hydrophobicity; More significant molecule diffusion; Limited thermal compatibility; Limited mechanical resistance.
PDMS [46]	Easy to fabricate; Elasticity and deformity; Optical transparency; Biocompatibility; Selective permeability.	Sorption of molecules; Gas permeability; Limited thermal compatibility; Limited durability; Difficult to integrate electronics.
PMMA [47]	Optical transparency; Easy to fabricate; Chemical compatibility; Biocompatibility; Durability.	Gas permeability; Sorption of molecules; Limited thermal compatibility; Difficult to integrate electronics; Relative stiffness.
HDPE [48]	Low cost; Easy to fabricate; Durability; Chemical compatibility; Electrical isolation.	Permeability; Sorption of molecules; Limited thermal compatibility; Difficult to weld; Relative stiffness.
LDPE [49]	Low cost; Easy to fabricate; Flexibility; Chemical compatibility; Electrical isolation.	Permeability; Sorption of molecules; Limited thermal compatibility; Difficult to weld; Relative stiffness.
Polyamide 6 [50]	Mechanical resistance; Dimensional stability; Chemical compatibility; Easy to fabricate; Biocompatibility.	Gas permeability; Limited thermal compatibility; Difficult to weld; Sorption of molecules; Difficult to weld.
SU-8 [51]	High resolution; Photolithography compatibility; Design flexibility; Chemical compatibility; Mechanically stable.	Difficult to fabricate; Long fabrication time; Gas permeability; High cost; Limited thermal compatibility.

## Data Availability

The data presented in this study are available upon request from the corresponding author.

## Supplementary Materials

The following supporting information can be downloaded at: https://www.mdpi.com/article/10.3390/mi15070873/s1. Table S1: A comparison table including 3 years of state-of-the-art showcasing LOD, dynamic range, and sensitivity of the microfluidics devices with their application [143,144,145].

## Abbreviations

2D	Two-dimensional
3D	Three-dimensional
CAD	Computer-Aided Design
CFD	Computer Fluid Dynamics
CIC	Cardiotoxicity-on-a-Cip
dLCSC	differentiated LCSC
DNA	Deoxyribonucleic acid
FDM	Fused Depositions Modeling
FE	Finite Element
Gr	Grashof Number
HDPE	high-density polyethylene
LCSC	lung cancer stem cells
LDPE	low-density polyethylene
LOAC	Lung-on-a-Chip
MI	Mixing Index
OoC	Organ-on-a-Chip
PCR	Polymerase Chain Reaction
PDMS	Polydimethylsiloxane
PMMA	poly (methyl methacrylate)
POCT	Point-of-care testing
Re	Reynolds number
SLA	Stereolithography
SLM	Selective Laser Melting
ToC	Tumor-on-a-Chip
