# Supplementary material for: Advances in Microfluidic Systems and Numerical Modeling in Biomedical Applications: A Review
Source: Micromachines (Basel). 2024 Jun 30;15(7):873. doi: 10.3390/mi15070873 (PMC11279158; doi:10.3390/mi15070873)
Supplement: Supplementary file 1 [file micromachines-15-00873-s001.zip › micromachines-3050003-supplementary.pdf]

Supplementary material

Title: Advances in Microfluidic Systems and Numerical Modeling in Biomedical Applications: A Review

Authors: Mariana Ferreira, Violeta Carvalho, João Ribeiro, Rui Lima, Senhorinha Teixeira, Diana Pinho

Table S1. A comparison table including 3 years of state-of-the-art showcasing LOD, dynamic range, and sensitivity of the microfluidics devices with their application.

Microfluidic device	Year	Limit of detection	Dynamic range	Sensitivity	Applications	References
**Device 1**	**2021**	**6 µmol/L**	**0 to 100 mmol/L**	**Electrochemical detection, in particular, offers excellent sensitivity**	**Analysis of viscous samples**	**[1]**
**Device 2**	**2022**	**0.044 ng m/L**	**0.05, to 10 ng m/L**	**81.4%**	**Point-of-care**	**[2]**
**Device 3**	**2023**	**47 pg m/L**	**0.01 ng m/L to 100 µg m/L**	**high sensitivity**	**Point-of-care**	**[3]**

References

[1] K. Pungjunun *et al.*, "Laser engraved microapillary pump paper-based microfluidic device for colorimetric and electrochemical detection of salivary thiocyanate", doi: 10.1007/s00604-021-04793-2/Published.

[2] E. Huang *et al.*, "Active droplet-array microfluidics-based chemiluminescence immunoassay for point-of-care detection of procalcitonin," *Biosens Bioelectron*, vol. 195, Jan. 2022, doi: 10.1016/j.bios.2021.113684.

[3] S. Boonkaew, K. Szot-Karpińska, J. Niedziółka-Jönsson, B. Pałys, and M. Jönsson-Niedziółka, "Point-of-care testing for C-reactive protein in a sequential microfluidic device," *Sens Actuators B Chem*, vol. 397, Dec. 2023, doi: 10.1016/j.snb.2023.134659.
